# Development of Nomophobia Profiles in Education Students through the Use of Multiple Correspondence Analysis

**DOI:** 10.3390/ijerph17218252

**Published:** 2020-11-09

**Authors:** Clemente Rodríguez-Sabiote, José Álvarez-Rodríguez, Daniel Álvarez-Ferrandiz, Felix Zurita-Ortega

**Affiliations:** 1 Department of Research and Diagnosis Methods in Education, University of Granada, 18071 Granada, Spain; clerosa@ugr.es; 2 Department of Pedagogy, University of Granada, 18071 Granada, Spain; alvarez@ugr.es (J.Á.-R.); ferrandiz98@correo.ugr.es (D.Á.-F.); 3 Department of Didactics of Musical, Plastic and Corporal Expression, University of Granada, 18071 Granada, Spain

**Keywords:** nomophobia, higher education, education, smartphone, multiple correspondence analysis

## Abstract

(1) Background: Nomophobia is a recent behavioural addiction phenomenon. The present study proposes the objective of determining levels of nomophobia in students of Education. In addition, it seeks to find evidence regarding whether cross-tabulating variables produces statistically significant differences and to examine whether the contemplated variables, together with nomophobia levels, can be used to generate a student profile. (2) Methods: A total of 510 students (M = 20.69 years) participated in this study. For the collection of information, we developed a Likert-type ad hoc scale of nomophobia. The quantitative data analysis programmes SPSS v.25 (IBM, Armonk, NY, USA), STATA.v.15 (StatCorp, Spring, TX, USA) and jamovi v.1.2 (The jamovi project, Sidney, Australia) were used to analyse information collected by the previously described scale. (3) Results: The study concludes the existence of three main levels of correspondence. The first is formed by students with a low level of nomophobia. It is associated with students undertaking the first year of a Master’s degree who are older than 24, and in this case, gender does not play a discriminating role. The second describes students with moderate nomophobia. It is associated with females, the degree titles of Pedagogy and Primary Education, undertaking the first or second year of degree study and ages of between 21 and 24. Finally, the third level of correspondence is formed by students with high nomophobia. It is related to the same characteristics as those previously mentioned but ages typically ranging between 17 and 20.

## 1. Introduction

Beginning in the last decade and motivated mainly by the technological advances that have taken place in society, young people are typically referred to as digital natives, whilst some older individuals are referred to as digital immigrants, as they have adopted this Information and Communications Technology (ICT) society [1,2]. ICT brought with it an accelerated increase in internet use, social networks, smartphones and videogames. This led to positive consequences and potential advantages that guarantee personal security, knowledge and enable health promotion and better communication [3,4,5]. Unfortunately, it also led to negative consequences, as it increases the power of loneliness and depression [6,7], internet addiction [8] and so on. This could have perverse repercussions for teaching and learning processes in young people [9] or lead to the appearance of harmful practices and conducts such as cyberbullying, sexting or cyber addiction [10].

The mobile phone has experienced a very fast expansion worldwide. It currently represents an important part of techno culture, given that smartphones have become definitively installed into the daily lives of individuals [4]. In-line with this, data provided by Digital Marketing Trends 2015 [11] showed that 97% of the world population was using mobile phones in the year 2015 and that the number of devices owned by individuals was increasing. Further, there was a greater number of telephones than computers in Spain, with devices being used for more than three hours every day.

Nomophobia must be understood as a recent phenomenon of behavioural addiction. It consists, essentially, of the consideration of individuals with a strong fear of not having their mobile telephone. This may be because they forgot it or because it ran out of battery, leaving individuals finding themselves at some point “incommunicado” [12]. Whilst it is true that this new phobia is not currently tacitly collected in the DSM-V (Diagnostic and Statistical Manual of Mental Disorders), it could be considered to fall within the realm of the so-called situational phobia that is included in this manual. However, this leads to serious problems in younger populations, as it is presented as a pathological fear of being disconnected from technology [13].

Abusive use by individuals of mobile devices can generate habits that are difficult to eliminate. This can result in negative effects with regards to stress levels and sleep [14], disturbance, economic problems and cognitive or antisocial disorders [15]. In this sense, there are several papers showing that smartphone addiction can have serious health consequences related, especially, to the fear of missing out and social anxiety [16,17,18]. Even from a theoretical perspective, problematic smartphone use is described as a multifaceted phenomenon that is manifested in a variety of dysfunctional ways, such as addictive use and antisocial behaviours. Young people form the group most likely to develop disruptive behaviours as they find themselves at a stage that is critical for defining their identity [19]. From a developmental perspective, it could be a problem that young people are those who most use technology [20], given the continuous changes they are experiencing at the neurological, physiological and psychosocial levels [21,22]. In this sense, some authors have indicated in their studies negative repercussions on academic performance and impingements on family relations and maturation processes [23].

There is a scarcity of instruments available to evaluate mobile phone addiction largely because it deals with a very recent pathology. Of the tools found in Spain, Beranuy et al. [24] validated the Questionnaire of Experiences Related to Mobile phones (Q-ERM), which is used for the evaluation of the degree of mobile phone addition, and [25] adapted and validated the Nomophobic Questionnaire scale (NMP-Q) developed by Yildirim and Correia [26] in adolescents. Similarly, this scale was also adapted by González-Cabrera et al. [27] in diverse Spanish cities.

The studies being conducted worldwide are steadily increasing in number. In this way, Chiu et al. [28] established a possible correlation between internet addiction and mobile phone addiction. Within a population of students in Paraguay, Matoza-Báez and Carballo-Ramírez [29] determined that young people aged between 18 and 24 years are at a greater risk of nomophobia. In the same vein, Dias et al. [30] analysed a Portuguese population, whilst Rosales-Huamani [31] studied young Engineering students in Peru. Both uncovered high levels of anxiety and compulsive mobile phone use.

With regards to the instruments available for the evaluation of nomophobia, the majority of consulted studies are based on the questionnaire of Yildirim and Correia [26]. They designed and validated an instrument composed of 20 items in order to measure nomophobia in university students in the United States. Bragazzi et al. [32] then adapted this to the Italian context, including just three factors in disagreement with versions of the questionnaire that was previously adapted to Spanish [27,33] and Chinese contexts [34,35]. Prior to this, the mobile phone addiction scale (MPAS) was employed in students from Taiwan [28].

We can also highlight various works on the factors of influence regarding nomophobia. The first was developed in Taiwan [36] and produced as the main conclusions the finding that neither gender, duration of mobile phone possession nor narcotic substance use were associated with smartphone addiction. Another study by Haug et al. [37] was conducted in Switzerland and indicated that smartphone addiction was more frequent amongst younger adolescents in comparison with young adults. Further, work carried out by Yildiz-Durak [38] in Turkey analysed a set of demographic variables and reached the conclusion that age is the most important predictor, by a long way, of suffering nomophobia to a greater or lesser extent. Finally, work carried out in India by Bartwal and Nath [39] with students of Medicine reported that gender and age were equally the most relevant elements when considering the prevalence of nomophobia.

Although it has been reasonably well-established that young people feel the need to be continually connected to their mobile phones, a solid theoretical basis cannot be found that supports this disorder; neither is there unanimity in the established evaluation instruments [40]. In consideration of this, and bearing in mind that it has been presented by [9] that, up until the date of writing, little empirical evidence exists relating problematic mobile phone use with potential behavioural addictions, the present work proposes the following objectives: (a) To determine the levels of nomophobia (low, medium or high) within a sample of students of Education Sciences, according to their individual scale ratings and the characteristics of the sample being analysed; (b) to examine whether a cross-tabulation of different demographic variables relating to students (gender, age range, degree title and degree year) shows statistically significant differences with regards to the variable describing levels of nomophobia and (c) to check whether the demographic variables previously discussed, together with the variable describing the nomophobia level, produce any type of correspondence that can generate a student profile describing a higher or lower level of nomophobia.

## 2. Methods

The methodological design used for the present research corresponds to a descriptive study and, more specifically, to a survey study based on administration of a Likert-type scale on nomophobia to a representative sample of university students.

### 2.1. Participants

The sample size of our research study was *N* = 510 students from the Faculty of Education Sciences at the University of Granada (mean age was 20.69 years) whose differential traits will now be presented. With regards to gender, 23.7% were male, compared to 76.3% who were female. Relative to degree year, first-year students made up the greatest percentage with 38.2%, second-year students made up 30.8%, third-year students formed only 6.5% of the sample and fourth-year students corresponded to 17.3%, whilst the remaining 7.3% were on the first year of a Master’s degree. In reference to degree title, 21.2% of the sample were undertaking Primary Education, 15.9% Infant Education, 33.7% Pedagogy and 22% Social Education, whilst 7.3% were Master’s students. Finally, in relation to age, 55.7% were aged between 17 and 20 years, 34.4% were aged between 21 and 24 years and, lastly, the remaining 9.8% were older than 24.

On the other hand, we must highlight that, for the calculation of sample size, the following parameters were taken as a starting point: *N* = 6000, *p* = *q* = 0.5 and 1-α = 0.95, with a sampling error of ±4% calculated from the formula for finite populations propose for Thompson [41]:

(1) $$n=\frac{N\times Z_{a}^{2}\times p\times q}{d^{2}\times \left(N-1\right)+Z_{a}^{2}\times p\times q}$$

Using the following online tool: http://www.berrie.dds.nl/calcss.htm. The calculated sample size of this formula was 546 students, while our sample size had, finally, *n* = 510 students, with a sample loss of 36 participants. Finally, the sampling approach used adhered to proportional stratified probabilistic sampling, taking the variable describing degree title as the only proportional stratum.

### 2.2. Research Variables

A total of five different variables were considered as variables for investigation. On the one hand, four of these corresponded to the individual characteristics of students, these being: (a) gender: males vs. female; (b) degree year: first, second, third or fourth year of a Bachelor’s degree and first year of a Master’s degree; (c) degree title: Infant Education, Primary Education, Pedagogy, Social Education and Master’s; (d) year: 17 to 20 years, 21 to 24 years and older than 24 years and one corresponded to (e) the level of nomophobia: high, medium and low.

### 2.3. Instrument for the Collection of Information and Reliability and Validity

For the collection of information, we developed a Likert-type ad hoc scale of nomophobia through the selection/adaptation of items from other previously standardised instruments that deal with this topic. This instrument is formed by diverse demographic variables of the students, these being: gender, age, degree title and degree year. Further, it includes 28 items with 4 response categories: never, sometimes, frequently and always. Reliability and validity have been considered since the classical test theory (CTT) due to the inability to calculate an item response theory (IRT) model (e.g., 2 parameters, graded response model, nominal response model and modified graded response model). This difficulty in the adjustment of the item response theory (IRT) model to noncognitive tests is consistent with what we found in other researches. One possible explanation for this difficulty is that this type of research has so far been developed mainly on tests designed with CTT and not with ITR, which makes it more difficult to adjust [42].

CTT analysis obtained corrected item-total correlations of *r* > 0.20 for all items, apart from items 13 and 26. This indicates good general discrimination of the items making up the scale (George and Mallery [43]). However, with the aim of consolidating the results obtained through the CTT, we also submitted the data obtained from the scale to an IRT analysis. To explain in detail, we developed a graded response model (GRM) using the program Stata v.15 (StataCorp, Spring, TX, USA). The GRM is an extension of the Logistic Model of two paremeters (LM2p) and it enables us to identify the ability of items to discriminate between levels of the latent trait Samejima [44]. It is held constant, with item difficulty being established at each “step” of the item or when a response moves from one response category to another. In other words, for a 4-point response scale, as found in the present case, we would have *k*-1 steps (b parameters), given that participant responses can move from (a) 1 to 2, (b) 2 to 3, or (c) 3 to 4. Thus, *k* is the number of response options for a given item. In this way, the model would have 3 b parameters or threshold parameters. The model is structured in terms of cumulative probabilities and differences between accumulated differences. The probability that a participant selects response category *k* or higher is given by:

(2) $$P_{ik}^{*}\left(\theta \right)=\frac{e^{-1.7a_{i}\left(\theta -b_{ik}^{*}\right)}}{1+e^{-1.7a_{i}\left(\theta -b_{ik}^{*}\right)}}$$

According to the criteria described by Baker [45], the results obtained (not presented here due to space limitations) indicated that estimated values for discrimination parameters (*a*) suggested between acceptable (*a* > 0.65) and high (*a* > 0.1.34) discrimination for most items, with values of between 1.03 a 1.24 being achieved. Very high (*a* > 1.69) discrimination was also shown only in the case of items 1, 12, 21, 22, 27 and 28. Further, only three of the 28 items overall obtained values *a* < 0.65 (items 13, 18 and 26). As can be seen, these outcomes largely coincided with the CTT results obtained. Thus, we can conclude that the items were generally able to discriminate between students with lower vs. higher mayor nomophobia when discrimination criteria were applied. With regards to the threshold parameters (*b*), these ranged between −2.01 (*b_3_* item 6) and 12.98 (*b_3_* item 26), although the majority of values were between −2 and 2. This enabled accurate measurements of the latent trait of nomophobia to be taken within a large range. The distance between *b_k_* values was large for 28 of the considered substances. All categories emerged as most likely for some aspect of the measured trait. This appears appropriate for this scale, given that selecting a response option of 4 or 3 requires a much higher level of nomophobia in comparison to 1 or 2. This indicates that the information provided by this scale was more accurate at higher levels of the trait.

We contemplated reliability in terms of internal consistency (given that we included only a single administration of the measurement instrument) through analysis of Cronbach’s alpha and obtained a value of α = 0.88. This can be considered a high value, which denotes that high reliability is enjoyed by the scale [46,47].

In addition, we calculated the value of Cronbach’s alpha for the whole scale should each one of the 28 items be individually eliminated. In this sense, values of the Cronbach’s alpha coefficients for the scale were seen not only to fail to improve the obtained value but actually make it worse (all values below α < 0.88) should the scale items constituting the instrument be individually eliminated. This means that the items contemplated by the present study are necessary and should be maintained by the instrument, given that their suppression would provoke a reduction in the consistency level of the scale [43].

In addition, we also calculated the composite reliability, as well as the average variance extracted (AVE) of each of the factors using the jamovi programme [48] and Excel spreadsheet. For this purpose, we relied on the standardised factor loadings of each item with its reference factor, as well as the residual covariances.

The results obtained are shown in the Table 1:

As can be seen in the immediately preceding table, the composite reliability values are all equal to or greater than 0.71, while the AVE values are equal to or greater than 0.34. In the case, of composite reliability, values greater than 0.70 are preferable [49], and all the values obtained meet that condition. Moreover, for average variance extracted values, at least, equal to or greater than 0.5 is acceptable [50]. In our study, that condition is met, except in factor 1.

With regards to validity, we estimated content validity and concurrent criterion validity. Content validity of the instrument sought to guarantee that the items making up the instrument measure the construct that they were developed to measure; in other words, that the instrument measures what it is supposed to measure. In order to certify this validity, we constructed a scale based on three previously standardised instruments. In accordance with this, we have to outline that 8 of the instrument items were taken/translated from the screening for new addictions questionnaire (DENA) [51]. Another 8 of the questionnaire items were previously utilised by Ordoñez et al. [52], and, finally, the 12 remaining items were acquired from the instrument described by Roberts [53]. This left a total of 28 items that were scalar in nature.

We also considered concurrent criterion validity whose usefulness resides in determining the degree of correlation that individually exists between each scale item and the overall scale itself. For this purpose, we implemented the corrected item-total coefficient correlation. The results obtained in this respect indicated the cases in which *r* < 0.30 (items 20 and 28). For this reason, we can agree with the contributions of Dawson [54] and Salkind and Frey [55], which found that the items individually measured the same thing as the overall scale and, in addition, in the same direction (all of the correlations were positive). Thus, we can assert that the scale demonstrates concurrent criterion validity.

Finally, we estimated the construct validity of the scale. For this purpose, we submitted the 28 items of the scale to the exploratory factor analysis, using the quantitative analysis program SPSS v.25. The estimates used to provide a starting point will now be introduced. Extraction method used: principal component with Kaiser Criterion (λ ≥ 1). Rotation considered: Varimax.

With regards to the statistics prior to development of the factor analysis, a determinant correlation matrix value of |A| = 0.0000935 was achieved. This is to say, close to 0 but not null. This shows that the correlation matrix is not a single matrix and that the linear equations associated to the matrix could have a solution. On the other hand, the value of the Kaiser-Meyer-Olkin (KMO) measure of global adequacy was 0.871. According to Fabrigar and Wegener [56] and Holmes Finch [57], this value can be considered worthy. Measures of individual sampling adequacy (MSA) obtained values of ≥0.80 for all cases, this also being higher than the minimum value of MSA = 0.5 [58]. These results endorse the suggestion that the items’ bivariate correlations are more important than the outcomes obtained for the partial correlations. In addition, results for the Bartlett test for sphericity obtained a value of χ^2^ = 4497.882 (df = 378; *p* = 0.000). This means that the correlation matrix obtained is not an identity matrix; in other words, it is not a matrix where the correlations found in the diagonal are perfect (between the items themselves) and are null elsewhere in the square.

In relation to the results of the exploratory factor analysis implemented, we highlighted the presence of 8 factors that, when taken together, explain σ^2^ = 64.45%. As we will see later, and following the considerations of Carmines and Zeller [59] and Reckase [60], the first factor extracted does not explain at least 20% of the variance explained by the factorial solution, which is why we cannot consider the presence of unidimensionality on the scale used; however, it can be considered the main factor of the factorial model obtained. The commonalities obtained pertaining to each one of the variables of the scale are all greater than h^2^ = 0.5. This denotes that each and every one of the commonalities obtained good representation in the resulting factor solution. With regards to the final factor solution, we should highlight that the factor loadings were saturated at r > 0.35 (at least 10% of explained σ^2^, practical significance criterion).

Given these precedents, we contemplated a first factor comprised of 9 items (explained σ^2^ = 14.01%) that seemed to have in common something we call prominence; that is, a behaviour that becomes important when it is deeply integrated into the daily life of surveyed participants. It coincided with factor 2 pertaining to abuse and difficulty in controlling abuse from the scale presented by Meyers et al. [58].

We also had a second factor comprised of 6 items (explained σ^2^ = 9.96%), all of which had in common something we can refer to as abstinence symptoms. It coincided with factor 1 (not being able to communicate oneself) from the scale described by Yildirim and Correia [26].

We also found a third factor composed of 4 items (explained σ^2^ = 9.30%) related with tolerance of mobile device use. As with the second factor, the third also coincided with factor 1, pertaining to tolerance and abstinence from the scale of Meyers et al. [58].

The fourth factor, for its part, was also organized according to 4 items (explained σ^2^ = 7.05%), all of which had something to do with what we can refer to as comfort and euphoria resulting from mobile device use (coincident with factor 4, pertaining to giving up comfort from the scale described by Yildirim and Correia [26]).

The fifth factor was constructed of 3 items (explained σ^2^ = 5.64%) that maintained an association with conflicts generated by mobile device use in the family setting. The sixth factor was configured by 2 items (explained σ^2^ = 5.52%) that were linked to conflicts produced by mobile device use in the sphere of friendships.

The seventh factor was configured by a single item (explained σ^2^ = 5.43%) and was similarly related with conflicts created by mobile phone use at a social level. Finally, an eighth factor was presented that was also formed by a single item (explained σ^2^ = 4.47%). This had something to do with engagement in dangerous behaviours and personal integrity, such as mobile device use whilst driving a vehicle. Factors 5, 6, 7 and 8 from our scale could be seen as being reflected in factor 3 from the scale described by Meyers et al. [58], which globally referred to problems brought about by excessive mobile device use.

However, in order to demonstrate that the questionnaire worked similarly on males and females with measurement invariance analyses, we calculated a confirmatory factor analysis (CFA) by the jamovi program through the adjustment for maximum likelihood (ML). The results obtained about fit measures of CFA female vs. male are shown in the Table 2:

Confirmatory factor analyses conducted with male and female samples supported an eight-factor structure. In this structure, the obtained factors presented generally satisfactory standardised factor saturations (not presented here due to space limitations), which further provided strong evidence of the existence of factor invariance between studied male and female university students. In this respect, we should highlight that, in relation to CFA, measures of fit can be generally considered for both genders, whilst also appearing to be well-adjusted in both genders. Firstly, upon examination of the incremental fit indices of the comparative fit index (CFI) and the non-normed fix index or Tucker–Lewis index (TLI), values of 0.90–0.95 were obtained for both genders. For this reason, a reasonable fit was considered [61]. Secondly, if we consider the absolute fit measure of the root mean square error of approximation (RMSEA), we can see that both genders obtained higher values than that dictated by the cutt-point criteria of 0.06 advised by Hu and Bentler [62]. Nonetheless, given that this value has a known statistical distribution, confidence intervals can also be calculated (lower-upper) from the level of 90%. In this way, if the lower interval is above 0.05 and the upper value lower than 0.08, we can consider the fit to be reasonably good [63]. Finally, if we consider the parsimony-based fit measure of the Bayesian information criterion (BIC), according to Raftery [64], we can conclude that both considered models are well-adjusted. This is shown through the fact that BIC values >10 denote consistent fit, and, in our particular case, these values are well above this limit.

### 2.4. Ethical Considerations

Study participants gave informed consent before commencing with any type of participation. Participation was entirely voluntary, and participants had the opportunity to withdraw study data at any stage during its development. Information was stored in a secure and protected database, and any information enabling the identification of participants was destroyed.

## 3. Data Analysis and Interpretation

We used the quantitative analysis program SPSS v.25 to analyse the collected information. A diverse analysis that was descriptive, inferential and multivariate in nature was implemented in order to address the proposed research objectives.

In order to determine the levels of nomophobia (low, medium or high) within the sample of students, we considered their individual classification in relation to the total obtained for each student and a series of cut scores. These cut scores referred to the three tertiles that can be inferred by dividing all of the scores, which ranged from a minimum of 28 and a maximum of 112, into three equal groups as shown in the Table 3.

On the other hand, we show a detailed descriptive analysis of the three groups formed from the incidence of nomophobia (taking, with reference, the total scale) as shown in Table 4.

**Figure 1:**
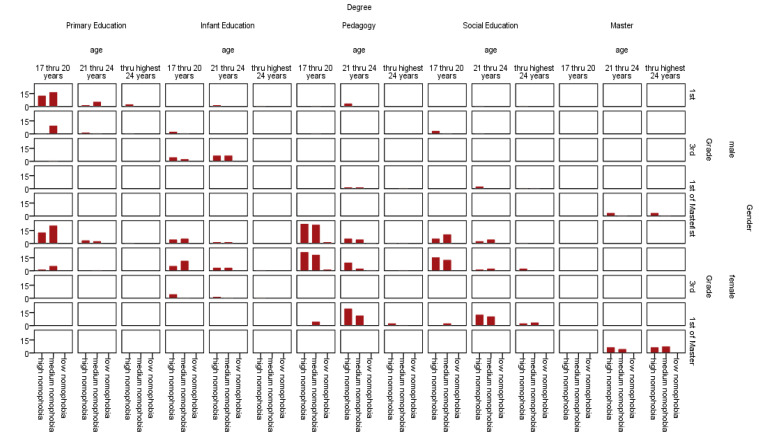
Burt table corresponding to the nested crossing of the variable levels of nomophobia by gender, by course, by degree and by age.

Bearing in mind the aforementioned classification, we present the levels of nomophobia amongst the students who formed the object of this research. Breaking the results down according to the demographic variable being considered—gender, degree year, degree title and age—within a single table in which these variables are nested, determined paths of correspondence are established. Definitively, we present a type of Burt table that is obtained when a multiple correspondence analysis (from now on, a MCA) is implemented. The one we present here is in Figure 1. 

Following the observations of the immediately preceding figure (Burt table), different correspondence paths immediately seem to be drawn at a merely descriptive level. In this way, we can appreciate how high levels of nomophobia appear to be more strongly associated with the female gender, those aged between 17 and 20 years, with the degree titles of Primary Education and Pedagogy and undertaking the first years of a degree (first and second). In contrast, the moderate and low levels of nomophobia appear to be related with other ages; that is to say, from 21 to 24 years and older than 24 years. In addition, they are linked to the degree titles of Infant Education and Social Education and to a Master’s degree and undertaking the final years of a degree course (third and fourth and first Master’s).

Nonetheless, this exclusively descriptive consideration must be empirically considered. With this aim (responding to research objective two), we developed four contingency tables in which we crossed-tabulated the four described variables (gender, degree year, degree title and age) with the nomophobia level, as shown in Table 5.

It can be appreciated that, except in the case of the variable describing gender where statistically significant differences were not reported (χ^2^ = 1.74; *p* > 0.05), the three remaining variables achieved χ^2^ statistic values that were all associated with a significant *p*-value (*p* < 0.05). These results contribute sufficient empirical evidence within the sample of participating students to support the continuing enquiry and the search for profiles capable of characterising a higher or lower incidence of nomophobia. For this objective (research objective three), we will implement the calculation of an MCA through the program SPSS, v.25. The MCA characteristics implemented are as follows: supplementary case range objects, normalisation method (principal variation according to the variables), convergence criteria (0.00001) and maximum iterations (100). The main results obtained are now shown below in Table 6.

The summary table of the model permits two inferred dimensions to be appreciated. Given that the eigenvalue considers the proportion of data in the model that is explained by each dimension, we can determine that factor or dimension one is the most important (inertia-associated λ_1_ = 2.395 and an explained σ^2^_1_ of 0.479). Further, it can be seen that factor or dimension two obtained an inertia-associated λ_2_ = 1.847 and an explained σ^2^_2_ of 0.369. This produces a total explained σ^2^_t_ of 0.848; in other words, almost 85% corresponds to an overall eigenvalue (λ_t_ = 4.242). For its part, the Cronbach’s α values obtained also indicate the extent of the correlations between the empirical variables (gender, degree year, degree title and age) that compose the latent variables (dimensions one and two). In our specific case, we can see that α_1_ = 0.728, whilst α_2_ = 0.573. We can, therefore, conclude that there is a greater correlation between the observed variables constituting factor one than those making up factor two.

**Figure 2:**
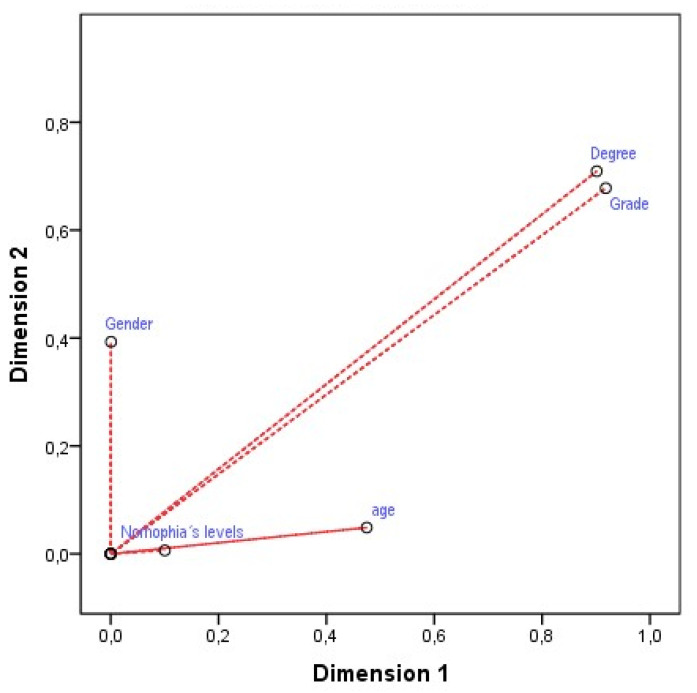
Discrimination measures of the variables.

With regards to the discrimination measures obtained for the different variables included in the analysis, we achieved significant results collected into Table 7 and Figure 2, which we will present next.

**Figure 3:**
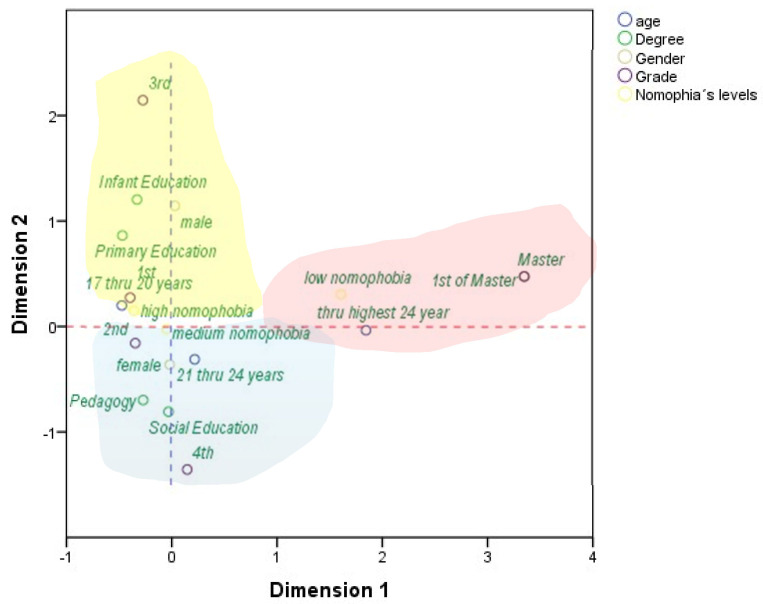
Joint plot of the category points.

As can be observed, we firstly highlighted that the variable pertaining to gender, in obtaining a parallel position to dimension two (x = 0; y = 0.410), becomes a good discriminating element of that dimension. This can also be said for the variables pertaining to the nomophobia level (x = 0.005; y = 0.101) and age (x = 0.475; = 0.055) with regards to dimension one. For their part, the variables pertaining to degree title (x = 0.902; y = 0.710) and degree year (x = 0.918; y = 0.666) achieved a bisector profile with regards to the equidistant origin, both for dimension one and dimension two. This converts them into discriminating variables of these two dimensions, and, further, as they are found further away from the origin, they possess greater explanatory power. Finally, we present the bio-spatial correspondence chart for the different levels of the contemplated variables, as shown in Figure 3.

As we can appreciate, the correspondence chart of the points from the various categories that form the five variables permits an organised structure to be drawn around three main correspondences. The first one would be composed (correspondence one) of students with low levels of nomophobia. It is associated with students undertaking the first year of a Master’s degree and who are older than 24 years of age, whilst gender does not play a discriminating role. The second correspondence is fulfilled by students with moderate nomophobia. It is more associated with females, the degree titles of Pedagogy and Primary Education, undertaking the first or second year of the degree course and individuals aged between 21 and 24 years. Finally, a third correspondence (correspondence three) would come formed by students with high levels of nomophobia. It is also more associated with females, the degree titles of Primary Education and Pedagogy, undertaking the first or second year of the degree course and being aged between 17 and 20 years.

## 4. Discussion of Study

The main aim of the present research sought to determine the levels of nomophobia within a sample of students of Education Sciences and to determine the potential correspondences between the parameters that formed the object of study. In relation to this, it must be indicated that the present study of nomophobia produced as results outcomes that are in agreement with those found in other consulted studies [33,65,66,67].

The majority of consulted research works indicate that high levels of nomophobia are associated with variables of a negative nature, such as anxiety, panic, impaired academic performance, lack of attention in class, impaired learning, etc. [67,68,69,70,71].

The results of the study indicated a stronger association of nomophobia with the female gender, within younger individuals and with the degree titles of Primary Education and Pedagogy. These data confirmed the results found in the studies of Hong et al. [36], Haug et al. [37] and Yildiz-Durak [38]. These pointed to a younger age and gender as relevant elements at the time of identifying and seeking to influence the prevalence of nomophobia.

In this sense, higher indices of socialisation correspond to the aforementioned age range, and mobile phone use constitutes one of the most socialising aspects of young populations [72,73,74]. As a result of this, a link between smartphone dependence and relationships with other individuals is generated. With regards to degree title, some authors indicated that the degrees of Social and Infant Education are highly vocational [75,76,77]. This implies a high capacity of attention towards these types of higher studies relative to the degrees of Primary Education and Pedagogy, which are not so specific or vocational. In a certain way, this enables students to prioritise other elements and could justify the results obtained in the present study.

As has been commented, individuals of a higher age and undertaking their final year of degree study acquire a larger degree of responsibility, and the levels of nomophobia decrease [78,79,80].

## 5. Conclusions

In this respect, the study determines three main correspondence levels. The first of these was formed by students with low levels of nomophobia and was associated with first-year Master’s students who were older than 24 years of age. Further, gender did not play a discriminating role. These results confirmed that which was indicated by Bragazzi et al. [81] and Yildirim et al. [82], who highlighted that age is a protective factor in the face of nomophobia. This is due to the fact that, as students grow older, they acquire a series of personal, family and working commitments that, in a certain way, replace the addiction to mobile phones. However, we will also see later on that, in profiles where levels of nomophobia are moderate or high, both gender and age play a much more discriminating role.

A second correspondence would be formed by students with moderate nomophobia that is found to be associated with the female gender, in addition to the degree titles of Pedagogy and Primary Education, undertaking the first or second year of degree study and being aged between 21 and 24. Finally, we contemplated a third correspondence formed by students with high levels of nomophobia that were also more associated with the female gender, in addition to the degree titles of Primary Education and Pedagogy, undertaking the first or second year of degree study and being aged between 17 and 20. Thus, sufficient empirical arguments exist for us to conclude that age is a key element with regards to nomophobia, as has been indicated in previous research works [33,34,35].

Finally, and in relation to the purpose at the outset of the present work, we can conclude that, effectively, in our particular case, we found three profiles of students of Education Science that were more or less associated with different levels of nomophobia. In these profiles, age, degree year and gender seem to contribute to a greater extent to the development of profiles outlining the levels of nomophobia. On the other hand, the variable describing degree title maintained a less influential role over the preparation of said profiles.

The data obtained showed the need to continue investigating other university populations and wider age ranges in order to establish correspondences with other psychological factors and establish more detailed profiles relating to the problem of nomophobia.

## 6. Limitations and Prospections of the Study

The limitations found in the present study are largely due to the realisation of a cross-sectional study in which data were collected at a specific time point. Another limitation is that the data cannot be generalised to the general university population, as only students of Education Sciences were analysed. It is also important to indicate that the ages involved were somewhat slanted, given that age ranges spanning only 27 years were included (the maximum age was 44 years vs. a minimum age of 17 years), and there existed a strong predominance of individuals with ages between 18 and 21 years.

Finally, another limitation of the study was the excessive presence of the female gender in the sample under investigation. However, this aspect is justified by the feminisation of the degrees studied at the facility under analysis.

## Figures and Tables

**Table 1:** Composite reliability and average variance extracted values of each factor.

Factor	Composite Reliability	AVE
F1	0.91	0.34
F2	0.88	0.56
F3	0.85	0.55
F4	0.76	0.52
F5	0.73	0.62
F6	0.71	0.57
F7	1	1
F8	1	1

**Table 2:** Fit measures of confirmatory factor analysis (CFA) females vs. males. CFI: comparative fit index, TLI: Tucker–Lewis index, RMSEA: root mean square error of approximation and BIC: Bayesian information criterion.

RMSEA 90%CI						
Gender	CFI	TLI	RMSEA	Lower	Upper	BIC
Female	0.911	0.912	0.0611	0.0611	0.0718	22,793
Male	0.903	0.901	0.0782	0.0678	0.0886	7247

**Table 3:** The score intervals forming each tertile, and the maximum, minimum and mean scores for each scale.

Levels	Range of Reported Scores	Max. S.	Min. S	Mean. S
High	>p_66_ = >84.33 (third tertile)	112	28	112 + 28/2 = 70
Medium	p_33_ to p_66_ = 55.67 to 84.33 (second tertile)			
Low	<p_33_ = <55.66 (first tertile)			

**Table 4:** Descriptive statistics of three groups of nomophobia (taking, with reference, the total scale).

	Nomophobia Levels			Statistic	Std. Error
Total Scale	High nomophobia	**Mean**		81.23	1.038
		95% Confidence Interval for Mean	Lower Bound	79.11	
			Upper Bound	83.36	
		Std. Deviation		5.685	
	Medium nomophobia	Mean		54.52	0.406
		95% Confidence Interval for Mean	Lower Bound	53.73	
			Upper Bound	55.32	
		Std. Deviation		8.718	
	Low nomophobia	Mean		35.33	0.443
		95% Confidence Interval for Mean	Lower Bound	34.40	
			Upper Bound	36.27	
		Std. Deviation		1.879	

**Table 5:** Results of the tables contingence.

Crosstabs	Chi-Square Test	df	Asymp. Sig. (2-Sided)
Gender by nomophobia’s levels	1.746	2	0.418
Grade by nomophobia’s levels	23.665	8	0.003 ***
Degree by nomophobia’s levels	27.269	8	0.001 ***
Age by nomophobia’s levels	7.657	4	0.045 *

* *p* < 0.05, *** *p* < 0.001. df: degrees of freedom.

**Table 6:** Summary of the model inferred from multiple correspondence analysis.

Dimension	Cronbach’s Alpha	Variance Accounted for	
		Total (Eigenvalue)	Inertia
1	0.728	2.395	0.479
2	0.573	1.847	0.369
Total		4.242	0.848
Mean	0.650 *	2.121 *	0.424 *

* Mean of the values obtained in dimensions 1 and 2.

**Table 7:** Discrimination measures of the variables.

Variables	Dimension		Mean
	1	2	
Gender	0.000	0.410	0.205
Grade	0.918	0.666	0.792
Degree	0.902	0.710	0.806
Age	0.475	0.055	0.265
Nomophobia’s levels	0.101	0.005	0.053
Active Total	2.395	1.847	2.121
